# Supplementary material for: Using Patient-Specific 3D-Printed C1–C2 Interfacet Spacers for the Treatment of Type 1 Basilar Invagination: A Clinical Case Report
Source: Biomimetics (Basel). 2025 Jun 17;10(6):408. doi: 10.3390/biomimetics10060408 (PMC12190520; doi:10.3390/biomimetics10060408)
Supplement: Supplementary file 1 [file biomimetics-10-00408-s001.zip › biomimetics-3642778-supplementary.pdf]

Supplemental Table S1. Detailed scan parameters for C-Spine protocol.

**Parameter**	**PCD-CT**
Scanner	NAEOTOM Alpha
Tube voltage (kV) / filtration	120/Standard
VMI (keV)	70
QRM (mAs)	176/162
Target $CTDI_{vol}$ (mGy)/Phantom	13.9/32cm, 12.8/32cm
Automatic exposure control	CareDose/CarekeV
Total collimation (mm)*	144×0.4
Pitch	0.8
Table speed (mm/s)	92
Revolution time (s)	0.50
Reconstruction kernel	Br44f/Br64f
Reconstruction Algorithm (strength)	QIR (3)
(Reconstruction) Slice Thickness/Interval (mm/mm)	3/3, 0.4/0.4
